# Correlation of Multi-drug Resistance, Integron and *bla*ESBL Gene Carriage With Genetic Fingerprints of Extended-Spectrum β-Lactamase Producing *Klebsiella pneumoniae*

**DOI:** 10.5812/jjm.8747

**Published:** 2014-02-01

**Authors:** Mitra Ashayeri-Panah, Mohammad Mehdi Feizabadi, Fereshteh Eftekhar

**Affiliations:** 1 Department of Microbiology, Faculty of Biological Sciences, Shahid Beheshti University, General Campus, Tehran, IR Iran; 2 Department of Microbiology, School of Medicine, Tehran University of Medical Sciences, Tehran, IR Iran

**Keywords:** *Klebsiella pneumoniae*, Beta-Lactamase, Genotyping, Integrons, Drug Resistance, Multiple

## Abstract

**Background:**

Some genetic and phenotypic variables are associated among distinct microbial populations.

**Objectives:**

The associations between multi-drug resistance (MDR) phenotypes, prevalence of antibiotic resistance integrons (ARIs), *bla*_SHV_, *bla*_TEM_ and *bla*_CTX-M_ gene carriage and genetic fingerprints of random amplified polymorphic DNA (RAPD), confirmed by pulsed field gel electrophoresis (PFGE), were investigated among extended-spectrum β-lactamases (ESBL)-producing nosocomial isolates of *Klebsiella pneumoniae*.

**Materials and Methods:**

Susceptibility of 35 ESBL-producing *K. pneumoniae* nosocomial isolates to 22 antimicrobial agents was determined. Integron carriage was detected using specific primers for intI1, intI2 and intI3 genes by PCR.

**Results:**

All isolates were resistant to piperacillin and susceptible to imipenem. MDR phenotype was observed in 91.4% of the isolates. Class 1 integrons were detected in 21 (60%) and class 2 integrons in 3 (8.57%) of the isolates. Two of the isolates carried both classes and none harbored class 3 integrons. Significant correlations were observed between resistance to aminoglycosides, fluoroquinolones and sulfonamides, and between genotype groups with carriage of ARIs, MDR phenotype and *bla*_SHV_ gene carriage. ARI carriage was also significantly associated with MDR phenotype.

**Conclusions:**

Our findings suggest the possible co-carriage of some *bla*_SHV_ genes and ARIs on the same plasmids harboring the MDR genes. Possible role of integrons in dissemination of ESBL-encoding *bla*_SHV_ genes among ESBL-producing *K. pneumoniae* nosocomial isolates may be inferred.

## 1. Background

*Klebsiella pneumoniae* is responsible for up to 10% of all nosocomial infections (1, 2). The importance of the organism in hospital settings has been increasing due to the emergence and progressive spread of multidrug resistance; specifically the extended-spectrum β-lactamase (ESBL)-producing strains (3). More than 600 ESBL variants have been described and the majority of them belong to the SHV, TEM and CTX-M families (http://www.lahey.org/studies/webt.htm) (3). Horizontal gene transfer due to mobile genetic elements such as insertion sequences, transposons and conjugative plasmids, mediates intra and interspecies dissemination of not only the genes encoding ESBLs but also other antibiotic resistance determinants which are likely to form part of an antibiotic resistance integron (ARI) (3-5).

Three classes of ARIs (classes 1, 2, and 3) have been historically involved in multi-drug resistant (MDR) phenotypes and are identified based on their respective integrase genes (5). Various typing methods have been applied to understand transmission patterns of resistance genes and management of nosocomial infections (6). We have previously developed an optimized RAPD-PCR protocol for genotyping *K. pneumoniae*, comparable to PFGE (7). To understand the associations between phenotypic and genetic characteristics of multi-drug resistant pathogens can be useful for reliable detection of these bacteria in epidemiological studies. Some reports have suggested associations between ESBL production and resistance to several classes of antibiotics, as well as *bla*_ESBL_ with ARI genes carriage in *K. pneumoniae* (4, 8).

## 2. Objectives

In this study, the association between MDR phenotypes, prevalence of ARIs, *bla*_ESBL_ genes and RAPD profiles were investigated among ESBL-producing *K. pneumoniae* nosocomial isolates. 

## 3. Materials and Methods

### 3.1. Bacterial Strains

**Figure 1.**
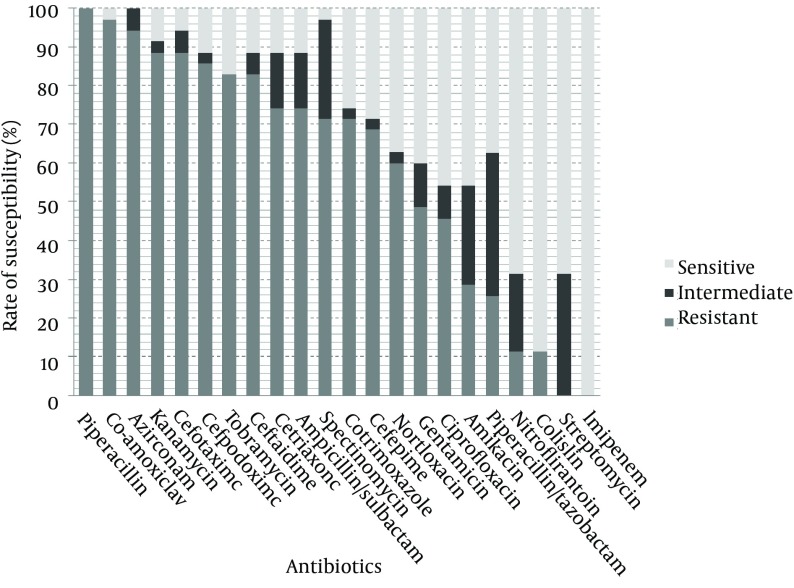
Antibiotic Susceptibility of ESBL-Producing Nosocomial Isolates of *K. pneumoniae* Measured by Disc Diffusion

Thirty five ESBL-producing nosocomial isolates of *K. pneumoniae* were randomly selected from a collection previously described (9). Bacteria were isolated from hospitalized patients at different wards of Labbafinejad teaching hospital, Tehran, Iran, during March 2008 – March 2009; subjects consisted of 23 (65.7%) male patients and 12 (34.3%) females. These isolates were recovered from urine (n = 23; 65.7%), trachea (n = 4; 11.4%), wounds (n = 4; 11.4%), blood (n = 2; 5.7%), sputum (n = 1; 2.9%) and unknown sources (n =1; 2.9%). ESBL production was confirmed using the phenotypic confirmatory test and susceptibility of the isolates to 22 antimicrobial agents (Himedia, India) shown in Figure 1, was determined by the disc diffusion method according to the CLSI criteria (10).

### 3.2. Screening for Antibiotic Resistance Integrons

Genomic DNA was extracted from overnight grown bacteria using High Pure PCR template Prep kit for Genomic DNA extraction (Roche Diagnostics, Mannheim, Germany). PCR amplification of classes 1, 2 and 3 integrase genes was performed in 25 µL reaction mixtures containing 30 ng DNA template, 0.4 mM of each dNTP, 150 µM MgCl_2_, 0.2 U Super Taq DNA polymerase (CinnaGen, Tehran, Iran) and 1 pmol of each primer (FazaBiotech, Tehran, Iran) as follows: Int1F; CCTCCCGCACGATGATC, Int1R; TCCACGCATCGTCAGGC, Int2F; TTATTGCTGGGATTAGGC, Int2R; ACGGCTACCCTCTGTTATC, Int3F; AGTGGGTGGCGAATGAGTG, Int3R; TGTTCTTGTATCGGCAGGTG) (11). 

Amplifications were performed in a Bioer TC25/H Thermal Cycler (Bioer Technology Ltd, Hangzhou, China) using the following program: initial denaturation at 95ºC for 5 minutes followed by 35 cycles of 1 minute at 94ºC, 1 minute at 60ºC and 1 minute at 72ºC with a final extension at 72ºC for 10 minutes. The amplified PCR products were resolved by electrophoresis in 1% agarose gels and visualized after staining with ethidium bromide.

### 3.3. Genetic Fingerprinting and Characterization of *bla*ESBL Genes

Genetic profiles of the isolates by RAPD, confirmed by PFGE have been reported in our previous article (7). Presence of *bla*_ESBL_ genes (*bla*_SHV_, *bla*_TEM_ and *bla*_CTX-M_) and the sequencing result for the isolates were also previously reported (9).

### 3.4. Statistical Analyses

To assess the strength and statistical significance of correlations between the studied variables including patient gender, type of specimen, antimicrobial susceptibility, MDR phenotypes (resistance to 6 or more antibiotics), carriage of ARIs, *bla*_SHV_, *bla*_TEM_ and *bla*_CTX-M_ genes and genotype grouping, and also measure the association between resistance to each of the aminoglycoside, quinolone and sulfonamide antibiotics, separate bivariate analyses were performed by use of the non-parametric Spearman's rank correlation test. To confirm the association between each pair of significantly correlated variables after factoring out the effect of other effective variables, partial correlation analyses were used. To interpret the results of correlation analyses, we considered correlation coefficients (r values) as well as the levels of significance (P values).

## 4. Results

The antibiotic susceptibility results are shown in Figure 1. As observed, all isolates were resistant to piperacillin followed by 97.1% resistance to co-amoxiclav, 94.3% to aztreonam, 88.6% to kanamycin and cefotaxime, 85.7% to cefpodoxime, 82.9% to tobramycin and ceftazidime, 74.3% to ceftriaxone and ampicillin/sulbactam, 71.4% to spectinomycin and cotrimoxazole, 68.6% to cefepime, 60% to norfloxacin, 48.6% to gentamicin, 45.7% to ciprofloxacin, 28.6% to amikacin, 25.7% to piperacillin/tazobactam and 11.4% to nitrofurantoin and colistin. All isolates were susceptible to imipenem. Streptomycin resistance was not observed but 31.4% of the isolates showed intermediate resistance. The most active antibiotic was imipenem followed by streptomycin, colistin and nitrofurantoin. Significant associations were observed between resistance to kanamycin, tobramycin, gentamicin, amikacin, norfloxacin, ciprofloxacin and cotrimoxazole (Table 1). Class 1 integrons were detected in 21 isolates (60%) and class 2 integrons in 3 isolates (8.57%). Two of the isolates carried both classes and none harbored class 3 integrons.

**Table 1.** Statistical Analyses Regarding the Associations Between Resistance to Aminoglycosides, Quinolones and Sulfonamides Among the ESBL-Producing Nosocomial Isolates of *K. pneumoniae*

Antibiotic classes	Antibiotic	Aminoglycosides										Quinolones				Sulfonamides	
		KM ^a^		TN ^a^		GM ^a^		AK ^a^		SM ^a^		NOR ^a^		CIP ^a^		TS ^a^	
		r ^b^	p ^c^	r	p	r	p	r	p	r	p	r	p	r	p	r	p
**Aminoglycosides**	KM	-- ^d^	--	0.789	0.1%	0.412	5%	0.363	5%	NS	NS	0.456	1%	NS	NS	NS	NS
	TN	0.789	0.1%	--	--	0.447	1%	0.460	1%	NS	NS	0.421	5%	NS	NS	NS	NS
	GM	0.412	5%	0.447	1%	--	--	0.436	1%	NS	NS	NS	NS	0.470	1%	NS	NS
	AK	0.363	5%	0.460	1%	0.436	1%	--	--	NS	NS	NS	NS	NS	NS	NS	NS
	SM	NS	NS	NS	NS	NS	NS	NS	NS	--	--	NS	NS	NS	NS	NS	NS
**Quinolones**	NOR	0.456	1%	0.421	5%	NS	NS	NS	NS	NS	NS	--	--	0.504	1%	0.410	5%
	CIP	NS	NS	NS	NS	0.470	1%	NS	NS	NS	NS	0.504	1%	--	--	NS	NS
**Sulfonamides**	TS	NS	NS	NS	NS	NS	NS	NS	NS	NS	NS	0.410	5%	NS	NS	--	--

^a^ Abbreviation: KM, kanamycin; TN, tobramycin; GM, gentamicin; AK, amikacin; SM, Streptomycin; NOR, norfloxacin; CIP, ciprofloxacin; TS, co-trimoxazole; NS, non-significant.

^b^ Correlation coefficients range between -1 (perfect negative relationship) and +1 (perfect positive relationship). A value of 0 indicates absence of any linear relationship.

^c^ Level of significance.

^d^ not available.

**Figure 2.**
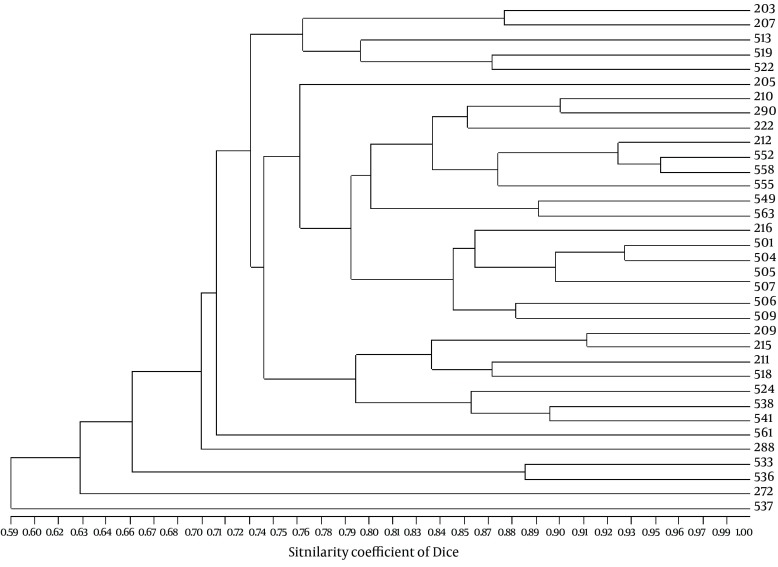
Cluster Analysis of the ESBL-Producing *K. pneumoniae* Nosocomial Isolates Based on RAPD Typing, Using the Dice Similarity Coefficient Isolate numbers are presented on the vertical axis.

Genetic profiles of the isolates by RAPD (Figure 2) which were confirmed by PFGE, showed six major clusters (a-f) on a similarity level of 70%, and 21 different groups on a similarity level of 85% (7). Characterization of *bla*_ESBL_ genes from our previous work showed that 27 isolates (77.1%) harbored *bla*_SHV_ genes including *bla*_SHV-12_, *bla*_SHV-5_ and *bla*_SHV-11_, 17 (48.6%) carried *bla*_TEM_ genes characterized as *bla*_TEM-1_ by sequencing, 16 (45.71%) carried *bla*_CTX-M-I_ which belonged to *bla*_CTX-M-15_ and 10 (28.57%) contained *bla*_CTX-M-III _characterized as *bla*_CTX-M-8_ (9).

Genotyping results were significantly correlated with carriage of ARIs (r = 0.700, P < 0.001; Spearman rank correlation test), *bla*_SHV_ (r = 0.742, P < 0.001) and MDR phenotype (r = 0.560, P < 0.001). Significant association was also found between ARI carriage and MDR phenotype (r = 0.398, P < 0.05). Although at the 95% confidence level, no significant association was observed between ARIs with *bla*_SHV_, *bla*_TEM_ and *bla*_CTX-M_ among the isolates. A positive association was found between class 1 integrons with *bla*_SHV-11_, *bla*_SHV-5_ and *bla*_SHV-12_ at a lower confidence level (r = 0.298, P < 0.1) (Table 2). Results of partial correlation analyses were also confirmatory (data not shown). No correlation was observed between the patient gender or specimen source with any of the genetic variables.

**Table 2.** Statistical Associations Between Genotypes, Carriage of Antibiotic Resistance Integrons (ARIs), *bla*_SHV_ Genes, and Multi-Drug Resistance (MDR) Phenotypes Among the ESBL-Producing Nosocomial Isolates of *K. pneumonia*

First Variable	Second Variable	r ^a, b^	P Value
**Genotype**	ARL_s_	0.700	0.1%
	*bla*_SHV_	0.742	0.1%
	MDR	0.560	0.1%
	Antibiotic susceptibility	-	NS ^a^
**ARLs**	*bla*_SHV_	0.298	0.097 (NS)
	MDR	0.398	5%
	Antibiotic susceptibility	-	NS
***bla*SHV **	MDR	-	NS
	Antibiotic susceptibility	-	NS

^a^ Abbreviations: r, correlation coefficient; NS, non-significant.

^b^ coefficients range between -1 (perfect negative relationship) and +1 (perfect positive relationship). A value of 0 indicates absence of any linear relationship.

## 5. Discussion

Infections due to ESBL-producing strains, have been most commonly reported regarding *K. pneumoniae *(3). ESBL encoding genes are usually located on plasmids which may also carry other antibiotic resistance determinants. Reports have suggested a close association between ESBL production and ciprofloxacin resistance in *K. pneumoniae* (8). Co-resistance with other classes of antibiotics such as fluoroquinolones, aminoglycosides, tetracyclines, chloramphenicol and sulfonamides are also widespread among ESBL producing strains (12). This may explain the significant associations found between resistance to aminoglycosides (kanamycin, tobramycin, gentamicin and amikacin) in this study. The same trend was observed for the association of resistance between norfloxacin with kanamycin, tobramycin, ciprofloxacin and cotrimoxazole. Similarly, resistance to ciprofloxacin and gentamicin were associated showing a relationship as a sign of co-carriage.

Bivariate correlation analyses followed by partial correlation analyses in order to distinguish between direct and indirect interactions, confirmed the results. Despite high heterogeneity observed among the isolates of this study, genotyping results were strongly correlated with carriage of ARIs and *bla*_SHV_ genes. Although almost all *K. pneumoniae* isolates carry chromosomal non-ESBL *bla*_SHV-1_, nearly all ESBL encoding *bla*_SHV_ genes found in *K. pneumoniae* are plasmid borne (13, 14). In this study, RAPD profiles were strongly correlated with the presence of *bla*_SHV_ genes suggesting that plasmid mediated *bla*_SHV-5_ and *bla*_SHV-12_ (the two prevalent ESBL encoding *bla*_SHV_ genes among our isolates) had some influence on RAPD patterns. Possible contribution of plasmid DNA to RAPD patterns was suggested in *K. pneumoniae* (15). However, Elaichouni et al. found no influence of plasmid DNA on the RAPD profiles in *Escherichia coli* and claimed that the amount of chromosomal DNA per cell in natural conditions inhibits observable plasmid amplification (16). The association of *bla*_ESBL_ genes with ARIs occurs when both form parts of complex integrons or are located on the same plasmid (4, 17).

We found a positive association between class 1 integrons and *bla*_SHV-11_, *bla*_SHV-5_ and *bla*_SHV-12_ at the confidence level of 90% (P < 0.1). Since genotyping results were highly correlated with the carriage of both ARIs and *bla*_SHV_, it could be concluded that ARIs and *bla*_SHV_ genes are carried on the same plasmids, or *bla*_SHV_ genes are located within ARIs at least among some of our isolates. Association between ARIs and *bla*_SHV-5_ as well as co-location of *bla*_SHV-12_ and a class 1 integron on the same plasmid have been reported (17, 18). However, other investigators have found a low rate of association between integrons and ESBL genes with the exception of *bla*_CTX-M-9_ (19).

Presence of plasmids that carry ESBL encoding genes as well as integron mediated antibiotic resistance has been reported among nosocomial isolates of *K. pneumoniae* (17, 19, 20). In most of these studies, ESBL encoding genes were located on plasmids but not within the integrons. Although most of the findings so far suggest contribution of integrons in the acquisition and transmission of resistance genes among bacteria, further investigations are needed to evaluate the involvement of other factors in transmission of linked resistance genes.
